# The Systematic Multiverse Analysis Registration Tool for defining multiverse analyses

**DOI:** 10.1098/rsos.250800

**Published:** 2025-10-29

**Authors:** Cassie Ann Short, Yusuf Coşku Inceler, Maximilian Frank, Andrea Hildebrandt

**Affiliations:** ^1^ Department of Psychology, Carl von Ossietzky Universität Oldenburg, Oldenburg, Niedersachsen, Germany; ^2^ Department of Psychology, Ludwig-Maximilians-Universität München, München, Bayern, Germany

**Keywords:** multiverse analysis, transparency, robustness, preregistration, documentation

## Abstract

Multiverse analysis is increasingly recognized as a systematic framework for assessing the robustness of scientific results across alternative defensible data processing and analysis pipelines. However, defining the multiverse, by identifying defensible combinations of options across multiple nodes in the analysis workflow, remains a cognitively and logistically demanding and complex task. Consequently, documentation of how multiverse analyses are constructed and the rationale behind decisions made is often incomplete, which risks the transparency and interpretability of robustness claims. The Systematic Multiverse Analysis Registration Tool (SMART) addresses this gap. SMART guides users from diverse scientific disciplines through the construction of multiverse analyses via a transparent, stepwise workflow. It guides the users through all defensibility and equivalence decisions and documents each decision made. This supports the creation of complete multiverses within user-defined criteria, with visual and numerical feedback to highlight potential errors along the procedure. The exportable documentation can be used for preregistration or included as supplementary material alongside the published manuscript to report uncertainty at the level of multiverse construction. By increasing the transparency, reproducibility and rigour in multiverse construction, and bringing cohesion to this procedure across multiverse analyses, SMART facilitates more interpretable robustness assessments and contributes to the broader goals of open and reproducible science.

## 1. Introduction

Low replicability of scientific findings has been reported across a range of disciplines [1–5]. In response, numerous large-scale initiatives have aimed to identify and address the underlying causes [6–8], and several contributors to low replicability have been identified, including low statistical power, fragmented theoretical landscapes, and heterogeneity in data processing and analysis procedures [8]. Central to the latter issue is the flexibility held by researchers to choose from multiple defensible options at several stages of the data processing and analysis workflow. This has been termed ‘researcher degrees of freedom’ [9]. Alternative options across multiple decision nodes,^1^ defined as specific points in the research workflow where a methodological choice must be made, that could be applied to the same dataset to answer the same research question, can be combined to create multiple defensible data processing and analysis pipelines, referred to as the ‘garden of forking paths’ [11]. Multiple studies across different scientific disciplines have reported that applying alternative pipelines to the same dataset to answer the same research question can lead to heterogeneous results [12–17]. Thus, when a result is reported following a single pipeline, the robustness of that result to alternative, equally defensible pipelines remains uncertain [18]. Moreover, because researchers rarely correct for the analyses they did not perform, this creates an invisible multiplicity problem [19], which increases vulnerability to type I errors as one of the potentially multiple results may reach significance by chance [20]. Uncertainty about the robustness of results to alternative defensible data processing and analysis pipelines is thus a central methodological challenge to replicability.

One promising solution to the challenge of unreported data processing and analysis uncertainty is multiverse analysis [21]. We use the term multiverse analysis [21] as an umbrella concept for the systematic exploration of multiple defensible analytic pipelines [10,22]. Rather than relying on a single data processing and analysis pipeline, multiverse analysis involves comprehensively and systematically computing multiple alternative defensible data processing and analysis pipelines and explicitly reporting the results across these variations [10]. Specifically, all stages in the workflow where multiple defensible options are available, called decision nodes in the following, are identified. Depending on the scope of the study, this may include decision nodes related to theoretical conceptualization, data processing, variable quantification and statistical analysis by model selection and parameter estimation [18]. By combinatorially generating all options across all decision nodes and removing those combinations deemed indefensible, the multiverse of pipelines is defined. All, or a representative sample of all [23,24], pipelines are then computed and the resulting estimates are reported as a distribution [25]. Thereby, multiverse analysis communicates the data processing and analysis uncertainty around the effect of interest and allows identification of the data processing and analysis decisions to which the effect is robust or not. Table 1 defines the core terms used in this manuscript, along with common synonyms used in prior work, and justifies our chosen terminology.

**Table 1.** Terminology used in this manuscript and corresponding synonyms across the literature.

term	definition	common synonyms across multidisciplinary literature	justification for use
multiverse analysis	systematic computation and reporting of results across multiple defensible data processing and analysis pipelines	specification curve [25], vibration of effects [26], multimodel analysis [27], manyverse [28], computational robustness analysis [15], many analysts approach [17], cooperative forking paths analysis [29]	most inclusive and increasingly adopted as an umbrella term across fields for a systematic and comprehensive robustness analysis approach [10,22]
decision node	a step in the workflow where multiple defensible options exist, and a methodological choice must be made	step [23], parameter [30]	emphasizes structure and avoids confusion with statistical model parameters [10]
option	one of the alternative defensible methods available at a decision node	strategy [23], parameter value [30]	emphasizes structure while avoiding confusion with higher-level strategies and avoids confusion with statistical model parameters [10]
pipeline	a complete sequence of selected options from raw data to output	forking path [11,29], specification [25]	emphasizes linear execution of decisions; aligns with much of the multiverse analysis literature

Multiverse analysis is gaining traction across several scientific disciplines as a comprehensive and systematic approach to robustness assessment, and conceptual frameworks have emerged to support its implementation. For example, a theoretical framework for a ‘principled multiverse’ has been proposed, in which only pipelines deemed equivalent based on an *a priori* criterion such as validity or precision of effect estimation are included, deflating the multiverse to substantively meaningful variation [31]. However, practical standards for defining equivalence, assessing it systematically and documenting the decisions transparently for reproducibility remain sparse. While step-by-step procedural guidance [10], computational tools [32,33], tutorials [34] and preregistration advice [35] provide support for conducting multiverse analyses, the process of transparently constructing a multiverse of pipelines and documenting the decision-making process remains without practical support, complex and inconsistently applied in practice.

Critically, the potential for multiverse analysis to explicitly report data processing and analytical uncertainty and increase transparency risks being undermined if the decision-making process used to construct the multiverse is not transparently documented. Without such documentation, reproducibility is compromised, the robustness report is susceptible to ambiguity and consequently potential bias, and pipeline selection remains open to strategic exploitation. To fully realize the benefits of multiverse analysis, the decision-making process used to construct the multiverse must itself be transparently documented and reproducible. However, preregistering a multiverse analysis poses unique challenges due to the sheer number and complexity of defensible pipelines that may be considered. Without a systematic approach to specifying the scope, identifying decision points and justifying the inclusion or exclusion of specific analytic paths, the risk of overlooked decisions, selective pipeline reporting and hidden multiplicity remains high. Existing preregistration platforms, while essential for timestamped archiving, offer limited support for documenting the complex and branching decision-making involved in constructing a multiverse analysis. As such, support to document the decision-making process, either prior to preregistration or to share as electronic supplementary material, is especially critical in multiverse analyses to ensure transparency and interpretability of robustness assessments.

An easy-to-follow practical tool with extractable documentation could support efficient and rigorous decision-making with greater simplicity, reduce bias and increase transparency. To address this, we created the Systematic Multiverse Analysis Registration Tool (SMART) to assist researchers from any empirical and quantitative scientific discipline to navigate the process of constructing a multiverse of defensible pipelines transparently and systematically and generate exportable reports of the full decision-making process. While researchers must still make the substantive decisions about defensibility, SMART reduces the logistical and cognitive burden of tracking and organizing those decisions by guiding users through a structured workflow, helping to ensure completeness and clarity of the procedure and its documentation. It prompts justifications for inclusion and exclusion decisions, visualizes the decision space to reduce omissions or redundancies, and enforces standardized terminology to improve clarity and interpretability. SMART also produces machine-readable outputs that support interoperability and reproducibility. SMART is designed to be statistically agnostic, allowing users to define the outcome metrics most appropriate to their research question and analytic framework, such as effect sizes, *p*-values, reliability estimates, or other summary statistics. This flexibility enables its use across diverse types of statistical analyses and scientific disciplines. The resulting comprehensive and structured reports are designed to be uploaded to existing preregistration platforms or shared as supplementary material alongside the subsequent publication, making the rationale behind pipeline inclusion and exclusion transparent, reproducible and open to review.

## 2. The Systematic Multiverse Analysis Registration Tool

SMART was developed using the Shiny toolkit [36] for the R programming language. Shiny simplifies the creation and deployment of web applications, enabling a wide range of users to access R-based tools online. The app is open source, the code is provided in a dedicated GitHub repository, and a step-by-step instructional video is available, featuring a running example that illustrates a full SMART workflow. An online version of the app is hosted by the Deutsche Forschungsgemeinschaft (DFG)-funded META-REP programme at https://www.apps.meta-rep.lmu.de/SMART/. The feedback received on a pilot version of the app during an informal user testing is available in the GitHub repository.

SMART is organized into two main sections: Multiverse 1.0, which focuses on the construction of all defensible pipelines, and Multiverse 2.0, which deflates Multiverse 1.0 to a principled multiverse based on a data-driven equivalence assessment. Each section contains subtabs for stepwise and structured progress. SMART records all decisions that are made and the justifications provided for them throughout the entire workflow. This record can be exported and used as a preregistration or shared with the published multiverse analysis to increase the transparency and reproducibility of the work.

### 2.1. The user interface

The SMART user interface is designed to support clear, structured navigation through the multiverse construction process. Specifically, the interface is divided into two main sections, one for Multiverse 1.0 and one for Multiverse 2.0, each containing modular steps labelled in the order in which they should be completed. Within each step, the user input generates colour-coded visualizations that are dynamically updated, supporting users to track their entries and progress in real time. This design ensures that the process remains transparent and manageable. The interface for each step of the procedure is described below.

Upon launching SMART, users are presented with the welcome page, which provides background information on the purpose of the tool and why it may be helpful. A second tab titled ‘Start’ requests a username for the purpose of uniquely labelling and organizing input and output files, enabling data import and extraction throughout the app procedure for the purposes of saving and returning. All data (username and pipeline decisions) are processed and stored in compliance with General Data Protection Regulation (GDPR) regulations. Users may save their progress at any time and return to their work later by using the extraction features and then uploading the extracted files in the ‘Start’ tab.

### 2.2. Multiverse 1.0

In Multiverse 1.0, all defensible pipelines are identified by stepwise completion of steps 1a–1d.

#### 2.2.1. Step 1a: define the multiverse space

With step 1a, the user defines the scope of the multiverse analysis and identifies, within that scope, the decision nodes that exist and the defensible options for each of those decision nodes.

*Plan the analysis*. Firstly, SMART provides three fields for users to document the analyses they intend to run on the multiverse results (e.g. descriptive or inferential analyses [37]) and whether a multiple testing correction will be applied to the multiverse results [38], and if so, to specify the planned correction method. Including this information ensures that any inferential adjustments are transparently preregistered.

*Specify the scope*. SMART prompts the user to specify the scope of the planned multiverse analysis using check boxes. Specifically, the user can select one or more of the following: ‘measurement’ (i.e. operationalization, measurement and/or collection of input and output variables), ‘preprocessing’ (i.e. preparation of data for analysis, such as cleaning and transformation), ‘model specification’ (i.e. specification of the statistical model) and ‘estimation methods’ (i.e. the method used to estimate parameters, such as ordinary least squares and maximum likelihood estimation). Additionally, under each of these categories, there is space for entering further details where the category does not fit the scope exactly. For example, a multiverse analysis may aim to report only the robustness of an effect to outlier handling decisions. In this case, the user can select ‘preprocessing’ and enter the specific element of preprocessing that represents the scope of the planned multiverse analysis, i.e. outlier handling. As such, SMART is flexible enough to be used for multiverse analyses in a wide range of scientific disciplines. Reporting this information at the outset clarifies the boundaries of the decision nodes for readers of the subsequent constructed multiverse analysis and provides the researcher with a clear and concrete starting point for constructing the multiverse.

*Method to identify defensible pipelines*. SMART prompts the user to specify the method used to identify defensible pipelines. A variety of methods has been used in the multiverse literature to date, including systematic literature reviews [39], individual or collaborative expertise [8] and crowdsourcing [40], which may influence the set of pipelines that are sampled and later considered for their defensibility. SMART prompts the user to select which of these methods or whether an alternative method is used and provides space for further detail, should this be necessary for greater specificity and transparency.

*List decision nodes and options*. At this point, the user is prompted to list all decision nodes and options that are possible within the specified scope, using the specified method. SMART provides clickable boxes to add decision nodes as many times as required, and within each node created, to add as many options as required. There is no limit to the number of nodes and options that can be added, and decision nodes with only one available option should also be added to ensure their inclusion in the subsequent construction of pipelines. Nodes and options are labelled using free text input rather than predefined labels, ensuring that the tool is flexible and can be used with high terminology precision in any scientific discipline. All decision nodes and options within the previously specified scope of the multiverse, regardless of their defensibility, should be added because defensibility is evaluated openly in the next step (1b). This is important for readers of the multiverse analysis to understand which nodes and options have been considered for defensibility and which have not. For example, if a reader believes that an option has been incorrectly excluded from the published multiverse analysis, they can check whether it was either never considered for defensibility or was considered but deemed indefensible. This helps to identify potential oversights and unintentional bias and greatly enhances transparency.

**Figure 1.**
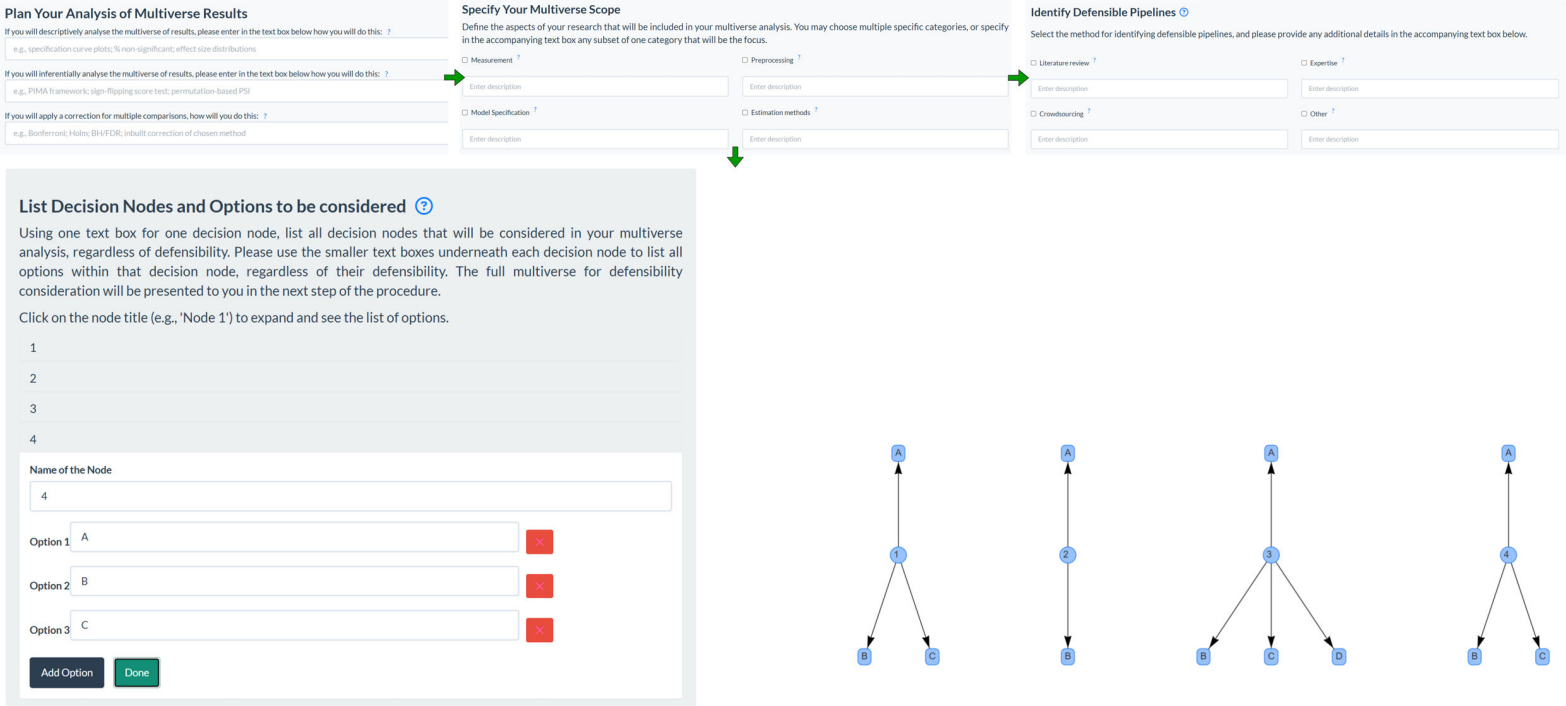
The sections of step 1a, where decision nodes and options can be added with direct reference to the specified scope, are visualized*.*

An accompanying visualization is generated on the right-hand side of the user interface, showing each decision node and the accompanying options. This figure is updated each time the user selects the button labelled ‘done’ within each node area. Presenting the nodes and options as a diagram makes it easier to identify any nodes or options that may have been overlooked and provides readers with an easier-to-digest presentation of the information alongside the textual lists. The sections of step 1a tab, are visualized in figure 1.

#### 2.2.2. Step 1b: define defensibility

Within step 1b, all the decision nodes and options identified in step 1a are to be classified as defensible or indefensible, thereby determining which decision nodes and options are to be advanced to the next step of pipeline construction. These are listed in the left-hand panel and presented as a figure on the right-hand side of the window. Each option at each decision node is to be considered independently for defensibility. Regardless of whether the defensibility of an option depends on other options along the pipeline, or on its position in the pipeline sequence, if the option is in any way defensible for the multiverse analysis (considering the study design, research question, data and epistemological framework), it should be classified as defensible here. Pipeline dependencies will be considered in a later step.

**Figure 2.**
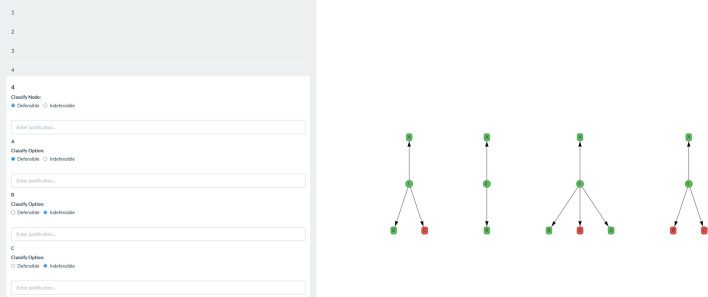
An example of a completed step 1b, where the defensibility classifications of each individual node and option are automatically colour-coded.

Each option must be classified as either defensible or indefensible by selecting the corresponding checkbox. Once classified, the node or option is automatically colour-coded as green (defensible) or red (indefensible) in the figure, providing a clear overview of the progress of decisions. A free entry text box below each node and option allows justification to be added where appropriate, such as if a generally defensible option is indefensible in the context of the research design or data. For example, replacing outliers with the group mean may be generally defensible but becomes indefensible in cases such as a binomial distribution. These justifications promote transparency and allow readers to evaluate the decisions made to arrive at the published multiverse analysis. All defensible nodes and options are carried forward to step 1c automatically. An example of a completed step 1b is presented in figure 2.

#### 2.2.3. Step 1c: create defensible pipelines

Within step 1c, all previously classified defensible nodes and options are displayed, and in this step, they are to be selected and organized sequentially to create defensible pipelines, considering any dependencies and order flexibility. All pipelines that are considered defensible should be constructed at this stage. This process allows readers to see which pipelines have been created from the pool of defensible options and which have not.

**Figure 3.**
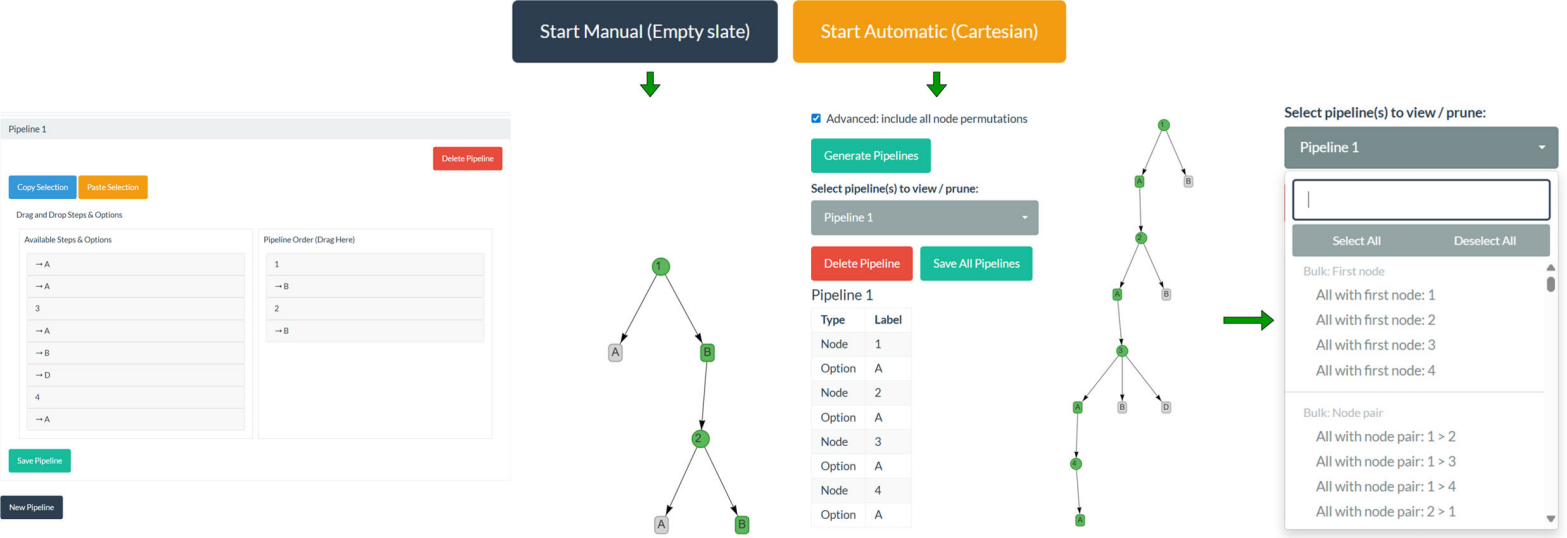
There are two options available in step 1c for combining defensible options into a defensible multiverse of pipelines.

Users can choose between two construction methods, depending on their multiverse analysis needs. Both available construction methods are visualised in figure 3. One method is to build the multiverse of pipelines from scratch, which allows users to manually define each pipeline, one by one, using a drag-and-drop feature from the pool of options that were classified as defensible in step 1b. This is particularly valuable when pipelines are derived from specific sources such as systematic literature reviews, where only a subset of the full decision space is relevant. The second method available enables users to automatically generate the Cartesian product of all defensible options across the decision nodes that were classified as defensible in step 1b. Users can then exclude any combinations they deem indefensible directly in the interface. This approach is especially useful when the entire decision space is initially plausible and large numbers of pipelines must be defined efficiently.

Following the first option, a new pipeline can be added by clicking on the ‘New Pipeline’ button, which will create ‘Pipeline 1’. Clicking on ‘Pipeline 1’ will open the construction interface, where all available options are presented in the left-hand box, organized under their respective decision node, and a blank canvas will appear in the right-hand box. The pipeline can be constructed by dragging and dropping options from the left box into the right box in the desired order. Each option should be preceded by its node when entered into the pipeline. Once a defensible pipeline is complete, clicking on ‘save pipeline’ will save it, and a notification will temporarily appear in the bottom right corner of the application window to confirm this. Further pipelines can be added by clicking ‘New Pipeline’ and repeating the process. All previously constructed pipelines remain accessible throughout. This process should be repeated until all defensible pipelines are constructed for Multiverse 1.0 (all defensible pipelines, prior to equivalence assessment). For simplicity and speed, sections of an existing pipeline can be copied and pasted into a new pipeline by selecting the desired sequence and selecting copy, then selecting paste at the current pipeline and continuing to build around it.

If users choose the automatic generation method, they can opt to generate the Cartesian product in one of two ways: (i) with decision nodes fixed in the order entered in step 1b (default), or (ii) with all possible permutations of decision node order (by ticking the box for the advanced option). In both cases, all possible combinations of defensible options across nodes, whether with a fixed or flexible node order, are created automatically. Users can then remove individual pipelines or apply bulk deletions based on specified criteria (e.g. removing all pipelines that begin with a certain decision node or that include a specific two-node sequence). This workflow allows users to rapidly generate a complete decision space and then systematically refine it by removing pipelines that are not relevant or defensible.

The application saves all pipelines and carries them forward to the next step (1d) automatically.

#### 2.2.4. Step 1d: your Multiverse 1.0

Step 1d displays a drop-down menu listing all the pipelines constructed in step 1c. Selecting a pipeline from the list will display the sequence of options in that pipeline as both a table and a figure. This allows the user to review the completeness of the pipeline and, when ready to proceed, to export all the pipeline information. To help users reflect on potential imbalances in their multiverse, SMART provides a frequency count showing how often each option at each decision node is used across all constructed pipelines. This feature allows users to identify whether certain options are unintentionally over-represented, which could bias the interpretation of robustness. By reviewing these counts before finalizing the multiverse definition, users can distinguish between over-representation that is substantively justified (e.g. due to logical or combinatorial constraints) and over-representation that may indicate an inadvertent bias.

**Figure 4.**
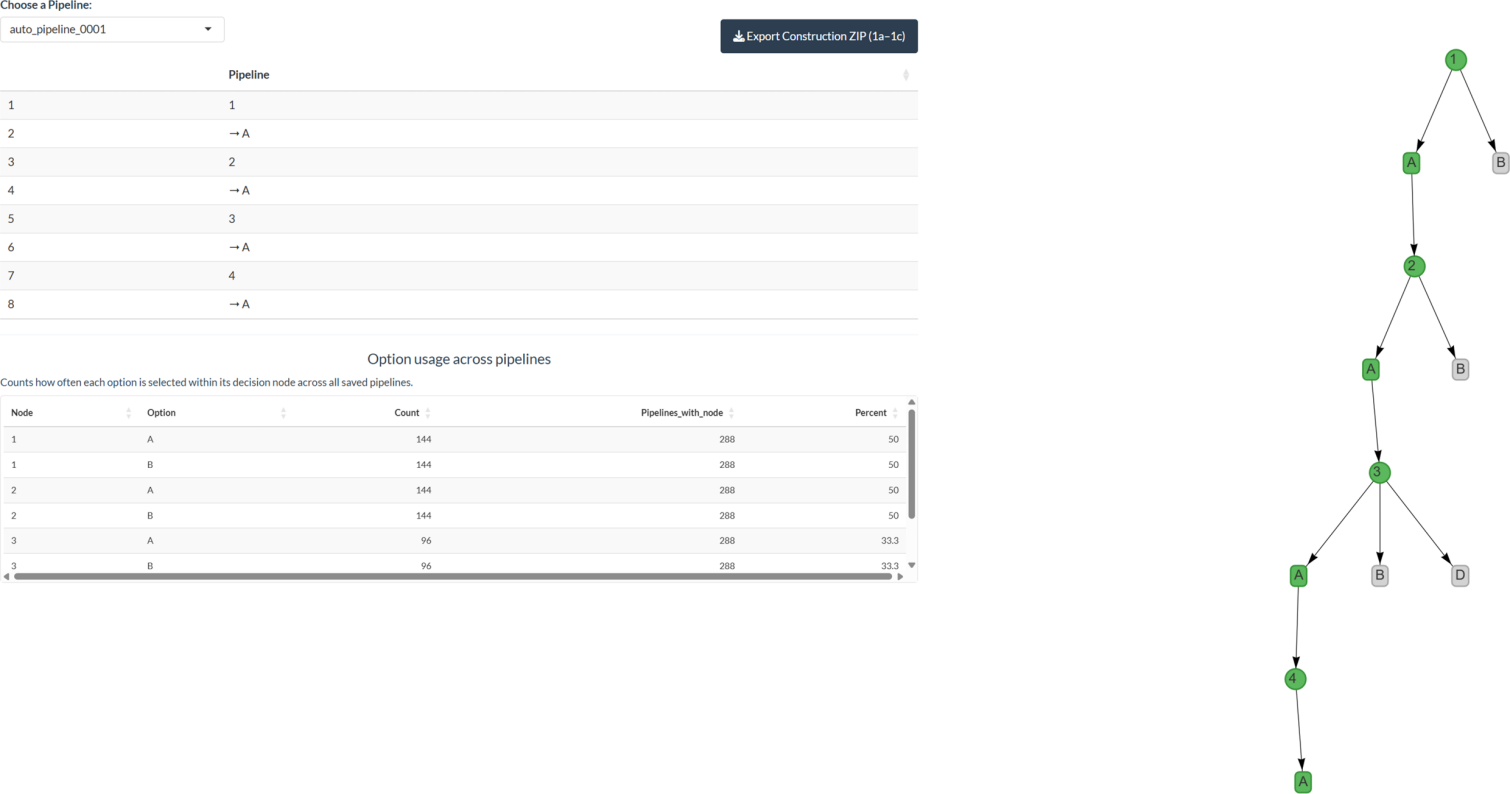
An example of the step 1d interface when pipelines have been defined and are ready to export*.*

Clicking on ‘Download Preprocessing Documentation’ will generate a .pdf file summarizing all the information entered and a tabular and visual network summary of each pipeline. Clicking on ‘Export Construction ZIP’ will generate and download a .zip file containing .csv files documenting the decision-making process from steps 1a, 1b and 1c, organized between steps. To ensure compatibility with step 2a of Multiverse 2.0, the folder structure and file names must remain unchanged if the user wishes to import their Multiverse 1.0 at a later date. This can be achieved by uploading the .zip folder to the respective field on the start subtab of the welcome page of the app. A visualization of the step 1d interface is provided in figure 4.

### 2.3. Multiverse 2.0

Multiverse 2.0 refines the set of defensible pipelines identified in Multiverse 1.0 into a principled multiverse [31] based on a user-defined equivalence criterion and threshold, and a data-driven assessment performed on a subset of participants. This involves computing Multiverse 1.0 on a partial sample or data from a pilot study to minimize computational demands, with the output being the equivalence criterion rather than the effect of interest that is later estimated to answer the research question. Those pipelines that produce a value of the equivalence criterion within the specified threshold are classified as equivalent, while those that produce a value outside the threshold are classified as non-equivalent. Only pipelines classified as equivalent are included in the principled multiverse (Multiverse 2.0). The Multiverse 2.0 section is intended for use by researchers who agree that all pipelines in a multiverse analysis should be equivalent according to a criterion relevant to the research question [31]. This stage consists of two steps.

#### 2.3.1. Step 2a: criterion for equivalence

The first part of step 2a requires the user to specify three decisions that define how equivalence is assessed to determine which pipelines constitute the principled multiverse. These are entered via three free-text fields to provide flexibility for different scientific disciplines. First, the equivalence criterion should be specified (e.g. standardized measurement error, intraclass correlation coefficient, or other metric deemed most relevant to the multiverse robustness assessment). Second, the threshold for equivalence in the specified criterion should be specified. This could be a point or interval threshold, where all pipelines resulting in a value above or below the point or within the interval are considered equivalent. Third, users should state whether they will perform the equivalence assessment on a proportion of their final sample of participants or using simulated data. If using the empirical data, the user should then specify in the accompanying box the proportion of the final sample of participants to be used for the equivalence assessment (e.g. 10%). When simulation is selected, users are prompted to briefly describe the simulation design and may optionally input a URL to the relevant code or documentation. Subsampling from real data enables equivalence assessment under the specific empirical conditions of the study, capturing realistic noise and structure, whereas simulation-based assessments allow pipeline performance evaluation across controlled, systematically varied scenarios. The latter approach may be preferred when sufficient empirical data is not yet available, in abductive frameworks, and when the aim is to provide a benchmark for equivalence for certain data structures.

All entries are saved and included in the final documentation generated at the end of the Multiverse 2.0 section automatically. After specifying these inputs, the user is prompted to compute the Multiverse 1.0 outside of SMART in their preferred analysis software (e.g. R, MATLAB, Python), estimating the specified criterion, on the specified subset of participants. Once completed, the user should return to step 2a to continue.

In the second part of step 2a, the user enters the results of the equivalence assessment. Specifically, each pipeline is selected one at a time from the drop-down list, and the corresponding value of the equivalence criterion is entered for each pipeline manually. We recommend using the pipeline index provided by SMART within the analysis software to avoid confusion. However, for verification purposes, the structure for each selected pipeline is displayed alongside the input field. Once the value is entered, the user classifies the pipeline as equivalent (‘Type E’) if the value falls within the predefined threshold, or as non-equivalent (‘Type N’) if the value falls outside of the predefined threshold. Automatically, a visual network generates on the right-hand side of the window, all inserted information is saved for export, and the pipelines classified as equivalent are carried forward to the next step.

#### 2.3.2. Step 2b: your principled multiverse

Step 2b is the final step, where the principled multiverse can be viewed and exported. All pipelines classified as equivalent in step 2a are included in the drop-down list and can be selected for individual inspection. Selecting ‘Download your Principled Multiverse PDF’ will download a .pdf file of all the imputed information from the Multiverse 2.0 section and the final set of Multiverse 2.0 pipelines.

## 3. Discussion

SMART is a practical tool to support researchers from different disciplines in constructing multiverse analyses in a systematic, transparent and reproducible way, improving ease, rigour and clarity in the decision-making process. Complementing existing conceptual frameworks [10,31] and implementation resources [32–35], SMART fills a critical gap by providing a user-friendly, practical platform for the often under-reported procedure of pipeline selection. It guides users through a stepwise workflow to identify study-specific defensible data processing and analysis alternatives, define pipeline combinations, refine them in a data-driven way using a study-specific equivalence criterion and justify each decision, including exclusions. SMART then generates structured, exportable documentation that clearly reports how pipelines were selected, evaluated and justified, supporting preregistration and transparency in published analyses. In doing so, SMART advances current practice by grounding the robustness assessments of multiverse analyses in transparent and clearly documented methodological decisions, thereby increasing reproducibility, methodological clarity and interpretability.

SMART was designed to be flexible enough to be applied across disciplines with different epistemological and methodological foci, while maintaining a standardized level of precision in the information it documents. First, it accommodates multiverse analyses focused on data processing, model specification, measurement, estimation, or combinations thereof, and multiple approaches to identifying options for consideration, such as systematic literature reviews, expertise, crowdsourcing and others, which can vary within and between disciplines [12–17]. It is also compatible with a wide range of statistical analyses, as users define the target metric for robustness assessment. Second, users can define their own equivalence criterion and threshold, allowing the app to adapt to study-specific research objectives, study designs and data types. Third, its modular structure supports different positions on the role of pipeline equivalence in multiverse analysis [10], providing both documentation of all defensible pipelines (Multiverse 1.0) and further, separate documentation following equivalence-based refinement (Multiverse 2.0), should this section be completed. This design ensures that the application is both flexible enough to accommodate a range of needs, yet structured and precise enough to support transparent, rigorous justification at every step. This flexibility is especially important given that the concept of defensibility may vary across confirmatory, exploratory and abductive research paradigms. Therefore, rather than imposing criteria, SMART provides users with a transparent structure for defining and justifying defensibility in their own context. For example, in confirmatory frameworks, defensibility may rely on theoretical constraints; in exploratory analyses, a broader range of options may be considered defensible; and in abductive approaches, defensibility may be guided by iterative refinement.

A key strength of SMART is its potential to reduce subjectivity in both the construction and appraisal of multiverse analyses by enhanced documentation. During construction, presenting the user with the full set of previously classified defensible options helps reduce the risk of inadvertently over-representing more familiar options in the final set of defensible pipelines. The application also promotes a data-driven approach to equivalence assessment by encouraging users to apply a pre-specified criterion to a subset of the data, treating equivalence as an empirical question rather than a subjective judgement. From an appraisal perspective, the exportable record of all decisions and justifications enables readers to independently review whether excluded options were considered and how inclusion and exclusion were determined. This level of transparency mitigates inadvertent bias and makes the decision space of the multiverse explicit and verifiable. Moreover, by formalizing multiverse construction, SMART has the potential to reduce variability across independently defined multiverses. As a result, it may help promote convergence in how multiverse analyses are defined and interpreted across studies, enhancing comparability and cumulative insights.

Beyond improving transparency and reproducibility, SMART also offers practical advantages over static documentation formats such as Word or LaTeX templates. Its interactive interface provides real-time visualizations that help users identify missing or inconsistently linked decisions, reducing the risk of oversight. By enforcing a structured format and consistent terminology, SMART minimizes ambiguity and supports interpretability for reviewers, replicators and meta-analysts. Moreover, the app generates machine-readable outputs, which are increasingly valuable for computational text analysis workflows for meta-analytic synthesis. These features make SMART a documentation tool that can identify errors prior to finalizing the multiverse and that can be integrated as a component of modern research infrastructure.

### 3.1. Limitation and future directions

SMART is well suited for small- to medium-sized multiverse analyses comprising up to a hundred pipelines [41], where manual construction and detailed justification are feasible. However, for large-scale multiverse analyses consisting of thousands of pipelines or more [15,16], manual specification becomes impractical. To address this, an automated sister application is under development that will allow users to input defensible options and their dependencies, from which the multiverse of defensible pipelines will be generated algorithmically. This tool will extend the principles of transparent and systematic construction to high-dimensional multiverse analyses, while maintaining the rigorous documentation provided by SMART. Nevertheless, the current app fills a critical methodological gap by providing researchers with an accessible platform to define their multiverse of pipelines where clarity and transparency are paramount.

SMART is open source to allow for community-driven development. As multiverse analysis continues to evolve methodologically across disciplines, the application can be extended or adapted to meet emerging needs. Future developments may include integration with knowledge spaces on data processing and analytical decisions in specific research fields [39], statistical analysis software, for specific data analytical approaches [42] and modules for visualizing decision tree structures. Unlike computational tools such as Boba [32] and Milliways [43], which support the execution and analysis of multiverse pipelines, SMART focuses on the pre-computational phase: the systematic identification, justification and formalized documentation of the analytic decision space prior to running analyses. In this sense, SMART is complementary to such tools, offering a structured foundation for transparent multiverse specification before statistical implementation. As researchers increasingly adopt multiverse analysis across disciplines, collaborative development is essential to promote cohesive standards and advance innovation.

## 4. Conclusion

In summary, SMART provides a structured, transparent and practical solution to one of the most complex and underdeveloped stages of multiverse analysis: defining the multiverse itself. By supporting and documenting stepwise, systematic decision making, rigorous justification and transparent equivalence-based refinement, it helps ensure that comprehensive robustness assessments are both interpretable and reproducible. As part of a broader movement towards open and reproducible science [4], SMART contributes a practical tool for more effectively communicating the uncertainty in reported results, thereby enhancing replicability.

## Data Availability

The app is open source, and all code is provided in a dedicated GitHub repository: https://github.com/cassiesh/MultiverseConstructionApp. A step-by-step instructional video is available: https://www.youtube.com/watch?v=f3_SiHl9tOA. The informal user-testing report is available: https://github.com/cassiesh/MultiverseConstructionApp. Supplementary material is available online [44].

## Footnotes

^1^
Terminology is used from [10]. Decision nodes refer to a specific step in the research workflow (pipeline) where the researcher must decide to either include or exclude the step, or a step where multiple alternative options are available for selection (e.g. outlier detection). Options refer to the alternative choices available at a given decision node (e.g. a method for outlier detection, such as ±3.29 s.d. from the group mean).
